# Growth mindset and grit as psychological resources in later life: age, socioeconomic status, and health patterning in the English Longitudinal Study of Ageing

**DOI:** 10.1093/geronb/gbag134

**Published:** 2026-07-11

**Authors:** Paola Zaninotto, Bran Knowles, Jasmine Fledderjohann, Alice Ashcroft, Andrew Steptoe

**Affiliations:** Department of Epidemiology and Public Health, University College London, London, United Kingdom; School of Computing and Communications, Lancaster University, Lancaster, United Kingdom; School of Social Sciences, Lancaster University, Lancaster, United Kingdom; Digital Heard Ltd, Lancaster, United Kingdom; Department of Behavioural Science and Health, University College London, London, United Kingdom; (Psychological Sciences Section)

**Keywords:** Learning behavior, Personality traits, Socioeconomic status

## Abstract

**Objectives:**

Growth Mindset and Grit have been proposed as key psychological resources for resilience and adaptation, yet their manifestation and social distribution in later life remain underexplored. This study examines the structure, distribution, and correlates of proxy indicators of Growth Mindset and Grit using the English Longitudinal Study of Ageing (ELSA).

**Methods:**

Proxy indicators reflecting learning behavior, personality traits, affect, and beliefs were used to derive 3 components of Growth Mindset (education-, personality-, and belief-based) and 2 components of Grit (affective- and personality-based). Multinomial logistic regression models were used to examine the correlates of Growth Mindset and Grit components, including age, socioeconomic status, health, and cognitive functioning.

**Results:**

Distinct distributional patterns emerged across components. Education-based Growth Mindset was concentrated in lower categories, whereas personality-, belief-, and affect-based components showed greater variability. Older age was associated with lower Growth Mindset, particularly in education- and belief-based domains, while associations with Grit were more nuanced, including a lower likelihood of low affect-based Grit among older adults. Higher educational attainment, employment, wealth, and better memory performance were associated with more favorable profiles across selected domains. Living alone and limiting longstanding illness were consistently associated with less favorable profiles.

**Discussion:**

Growth Mindset and Grit-related proxy indicators were socially patterned and showed distinct associations across belief, personality, affective, and behavioral domains in later life. The differing distributions observed across these domains highlight the importance of distinguishing between opportunity-constrained behavioral indicators, such as formal learning, and more dispositional characteristics when studying psychological resources in older populations.

Psychological resources that support learning, motivation, and persistence are increasingly recognized as important for understanding how individuals adapt to challenges across the life course. In later life, when opportunities, constraints, and vulnerabilities become more heterogeneous, such resources may be particularly relevant for explaining variation in engagement, resilience, and well-being among older adults. Two constructs that have attracted growing attention in this context are growth mindset and grit, which reflect orientations toward change, effort, and long-term goal pursuit. Despite their theoretical relevance to aging, however, evidence on how these constructs are distributed and patterned across the older population remains limited.

A growth mindset refers to the belief that abilities and personal characteristics are malleable and can be developed through effort and learning (Dweck, 2006). Alongside a growth mindset, the construct of grit has been proposed as a related psychological resource reflecting perseverance of effort and consistency of interests over time (Duckworth, 2016). Whereas a growth mindset concerns beliefs about the possibility of change, grit captures sustained effort toward long-term goals. In later life, grit may be particularly relevant for understanding how older adults maintain engagement and adapt to age-related challenges, although its role in aging remains under-examined.

Both Growth Mindset and Grit have conceptual links with broader psychological constructs that have received considerable attention in aging research. Growth Mindset shares similarities with perceived control and self-efficacy, which have been associated with healthier aging, better cognitive functioning, and greater resilience in later life (Lachman & Agrigoroaei, 2010). Grit overlaps with personality traits such as conscientiousness and openness to experience, which have been linked to health behaviors, wellbeing, and longevity (Kern & Friedman, 2008). While grit is strongly associated with conscientiousness, it has been theorized to differ in its emphasis on the sustained pursuit of long-term goals and the consistency of interests over time (Duckworth, 2016), although substantial empirical overlap has been reported (Credé, 2018; Credé et al., 2017).

While most research has focused on educational settings, growth-oriented beliefs may be particularly salient in later life, where age-related stereotypes and expectations of decline could shape attitudes toward learning, adaptation, and self-improvement (Dweck & Yeager, 2019; Heslin et al., 2021). Existing studies in older adults—largely cross-sectional and based on modest or regionally specific samples—suggest that growth mindset is associated with health behaviors, cognitive performance, and indicators of successful aging. Intervention studies further indicate that growth-oriented beliefs remain malleable in later life. For example, older adults participating in multi-skill learning programs have shown cognitive gains associated with individual differences in growth mindset (Sheffler et al., 2023), and structured interventions designed to foster growth-oriented attitudes have demonstrated improvements in cognitive functioning (Leanos et al., 2023). However, such studies typically involve selected volunteers and are not designed to characterize population-level distribution.

Research on grit in older adulthood has largely relied on cross-sectional survey designs, often using self-report measures in relatively small or regionally specific samples. For example, a study examined grit and successful aging in a community-based sample of 315 older adults in Korea (Kim & Lee, 2015), reporting positive associations between perseverance and successful aging indicators. A US study using a sample of 185 older adults (Rhodes & Giovannetti, 2022) found that grit was associated with multiple domains of physical, emotional, and social functioning. Fleck et al. (2024) similarly reported associations between grit and health outcomes in older adults. While these studies consistently suggest that grit is relevant to aging-related outcomes, their designs do not allow for an assessment of how grit is socially patterned across the full spectrum of later-life experiences at the population level.

Taken together, the existing literature provides important but partial insights. Evidence remains limited from large, nationally representative aging cohorts that can situate growth mindset and grit within broader social, health, and cognitive contexts. Large population-based studies offer an opportunity to examine whether these psychological dispositions are socially patterned according to socioeconomic position, health status, and cognitive functioning.

Socioeconomic position, health status, and cognitive functioning have been consistently associated with psychological resources relevant to adaptation and resilience in later life (Lachman & Agrigoroaei, 2010). Examining whether Growth Mindset and Grit show similar social and health patterning may therefore provide insight into how these resources are distributed across older populations.

The present study uses data from the English Longitudinal Study of Ageing (ELSA) (Steptoe et al., 2013), a nationally representative cohort of older adults in England, to operationalize theory-informed proxy indicators of growth mindset and grit, and to examine how these constructs are distributed across individual characteristics in later life. By providing a population-level perspective, this study extends prior research based largely on smaller or more selective samples and contributes to understanding the social and health patterning of growth-oriented psychological resources in older adulthood.

## Method

### Data

The data used in this study come from the English Longitudinal Study of Ageing (ELSA), an interdisciplinary ongoing cohort study of older adults aged 50 years and older living in private households in England (Steptoe et al., 2013). The study began in 2002 with a sample of approximately 12,000 individuals (response rate 67%). Data are collected biennially using face-to-face personal interviews, self-completion questionnaires, and health visits (every four years). The sample is periodically refreshed with new participants to ensure the study remains representative of those aged 50 and over. Details of the survey’s sampling frame, methodology, and questionnaires can be found at www.elsa-project.ac.uk. For this study, we used data from Wave 5 (2010–11), the first wave in which all indicators required to construct the Growth Mindset and Grit proxy measures were available simultaneously. Because some components were not collected consistently across waves, comparable longitudinal measures could not be constructed, and analyses were therefore restricted to Wave 5. A total of 9,090 participants completed the survey in Wave 5.

ELSA was approved by the London Multicentre Research Ethics Committee (MREC/01/2/91). Informed consent was obtained from all participants. All data are available through the UK Data Service (SN 5050).

### Measures

Growth mindset and grit were operationalized using theory-informed proxy indicators derived from personality, belief, behavioral, and affective measures available in Wave 5 of ELSA. Items were coded so that higher values consistently reflected stronger growth-oriented or grit-related tendencies before being standardized and combined into composite scores. Continuous composite scores were created by averaging standardized items and categorized into low (< −0.5 *SD*), moderate (−0.5 to 0.5 *SD*), and high (>0.5 *SD*) groups (see Supplementary Material for full description and Table S1 for full item wording). Categorization was used to facilitate interpretation and comparison across the different proxy indicators and to distinguish between relatively low, moderate, and high levels of each component in the absence of established thresholds.

#### Growth mindset personality

Trait-based growth mindset was derived from five items (Curious, Imaginative, Intelligent, Creative, and Broad-minded) from the Midlife Development Inventory (MIDI) (Lachman & Weaver, 1997), reflecting openness to change and intellectual engagement.

#### Growth mindset beliefs

Growth-oriented beliefs were operationalized using three CASP-19 items (Hyde et al., 2003) capturing perceived opportunity, future orientation, and willingness to engage in new experiences.

#### Growth mindset adult learning

Behavioral engagement in growth-oriented learning was measured using participation in formal education or training across Waves 4 and 5, categorized according to degree of engagement over the preceding four years.

#### Grit personality

Personality-based grit was derived from five MIDI items (Hardworking, Responsible, Calm, Organized, Active, and Thorough) reflecting perseverance, focus, and diligence.

#### Grit affect

Affect-based grit was constructed from five positive affect items (Determined, Hopeful, Attentive, Interested, and Inspired) from Watson et al. (1988), reflecting motivational engagement and sustained effort.

The components are explored separately because they are intended to capture distinct domains of Growth Mindset and Grit rather than indicators of a single latent construct. Growth-oriented and perseverance-related tendencies may be expressed through beliefs, personality characteristics, affective dispositions, or behavioral engagement, and these domains may not necessarily co-occur within individuals. Examining the components separately, therefore, allows us to explore potential heterogeneity in how these psychological resources are manifested in later life. Cronbach’s alpha coefficients for the multi-item Growth Mindset and Grit proxy indicators are reported in the Supplementary Material (Table S2). Internal consistency was acceptable for the Growth Mindset Personality (α  =  0.73), Growth Mindset Beliefs (α  =  0.76), and Grit Personality (α  =  0.75) components, and high for the Grit Affect component (α  =  0.88). Cronbach’s alpha was not calculated for the adult learning component because it was not constructed as a multi-item scale.

### Individual characteristics

To examine the social, socioeconomic, health, and cognitive correlates of Growth Mindset and Grit in later life, we included a range of characteristics, measured in Wave 5, that have previously been associated with psychological resources relevant to adaptation, resilience, and healthy aging. Sociodemographic characteristics comprised age, sex (male or female), living arrangement (alone vs not alone), ethnicity (White vs Non-White), age finished full-time education (14 or under, 15 to 18, 19 or over), employment status (in work, retired, other), wealth tertiles (measured as the sum of all physical wealth minus debts), and urbanicity (living in an urban or rural area). Health was assessed using a question on self-reported limiting longstanding illness asking whether the participant “has any long-standing illness, disability, or infirmity that has troubled [them] over a period of time or is likely to affect [them] over a period of time.” Participants responding affirmatively were classified as having a limiting, longstanding illness. We also included a cognitive function score derived from a memory assessment. Memory was evaluated using a word-list learning test, in which participants were presented orally with a list of ten words to recall immediately and after a short delay (Zaninotto et al., 2018). The overall memory score ranged from 0 to 20.

### Statistical analysis

The analytical sample consisted of individuals who completed the main interview and the self-completion questionnaire in Wave 5 (8,075 out of the 9,090 total participants in that wave, 89%). Complete-case analysis was carried out, and we therefore selected people with valid data on all variables of interest (a total of 7,333 out of 8,075, 91%). Multinomial logistic regression models were fitted separately for each Growth Mindset and Grit component. The outcome variables were the low, moderate, and high categories for each component, and all socio-demographic, socioeconomic, health, and cognitive characteristics were entered simultaneously into the models.

Descriptive statistics and regression models were weighted for non-response using inverse probability sampling weights. All analyses were conducted in Stata v19.

We conducted a sensitivity analysis in which the Growth Mindset personality, Growth Mindset beliefs, Grit personality, and Grit affect components were analyzed using their underlying continuous scores rather than categorical outcomes.

## Results

### Descriptive statistics

Table S3 shows the distribution of growth mindset and grit components. Growth mindset based on adult education was heavily skewed toward the low category, whereas personality and belief components were more evenly distributed. Grit components showed a centrally skewed distribution peaking in the moderate category and broadly balanced distributions in the low and high categories. Table S4 indicates age gradients across most components, particularly for adult education-based growth mindset, where participants in the low group were substantially older than those in the high group. Age differences were more modest for personality and affect-based components.

#### Adult education component of growth mindset

Table 1 presents the relative risk ratios (RRRs) and 95% confidence intervals (CIs) from a multinomial logistic regression model examining simultaneously sociodemographic and health-related correlates of the growth mindset-oriented adult education component.

**Table 1:** Correlates of the growth mindset-oriented adult education component, England 2008–2012.

Variables	Low vs moderate		High vs moderate	
	RRR (95% CI)	*p*	RRR (95% CI)	*p*
**Sex**				
Men	Ref.		Ref.	
Women	0.79 (0.67–0.92)	.004	0.81 (0.64–1.02)	.075
**Age group (years)**				
50–59	Ref.		Ref.	
60–69	1.33 (1.11–1.60)	.002	0.74 (0.58–0.95)	.020
70–79	2.08 (1.62–2.66)	<.001	0.36 (0.23–0.57)	<.001
80+	2.67 (1.72–4.14)	<.001	0.29 (0.12–0.68)	.004
**Ethnicity**				
White	Ref.		Ref.	
Non-White	0.81 (0.51–1.28)	.365	0.83 (0.42–1.66)	.600
**Living alone**				
No	Ref.		Ref.	
Yes	0.80 (0.65–0.97)	.025	0.83 (0.61–1.13)	.238
**Education**				
≤14 years	Ref.		Ref.	
15–18 years	0.86 (0.58–1.27)	.438	0.72 (0.39–1.33)	.289
≥19 years	0.48 (0.32–0.72)	<.001	0.59 (0.31–1.12)	.109
**Employment status**				
Retired	Ref.		Ref.	
In paid employment	0.25 (0.19–0.33)	<.001	1.10 (0.69–1.76)	.694
Other^a^	0.57 (0.44–0.75)	<.001	1.15 (0.72–1.86)	.553
**Wealth tertile**				
Lowest	Ref.		Ref.	
Middle	0.77 (0.62–0.94)	.012	0.83 (0.62–1.11)	.209
Highest	0.61 (0.49–0.75)	<.001	0.60 (0.44–0.82)	.001
**Area**				
Urban	Ref.		Ref.	
Rural	1.15 (0.97–1.37)	.109	1.08 (0.84–1.39)	.546
**Limiting illness**				
No (ref)	Ref.		Ref.	
Yes	1.26 (1.05–1.51)	.011	1.06 (0.80–1.39)	.686
**Memory score (continuous)**	0.93 (0.91–0.95)	<.001	0.97 (0.94–1.01)	.110

*Note.* RRR = relative risk ratio. Estimates adjusted for nonresponse sampling weights.

a Permanently out of work, temporarily out of work, and looking after family.

##### Low versus moderate growth mindset adult education component

Women were less likely than men to report a low personality-based growth mindset (RRR = 0.79, 95% CI: 0.67–0.92, *p* = .004). Age showed a strong graded association, with progressively higher relative risks of low growth mindset at older ages compared with those aged 50–59 (RRR = 1.33 for ages 60–69; 2.08 for 70–79; and 2.67 for 80+, all *p* < .01). Individuals living alone were less likely to report a low growth mindset than those not living alone (RRR = 0.80, 95% CI: 0.65–0.97, *p* = .025). Higher educational attainment was protective: participants who completed education at age 19 years or older were substantially less likely to report a low growth mindset compared with those who left school before age 14 (RRR = 0.48, 95% CI: 0.32–0.72, *p* < .001). Employment status was a strong predictor: individuals in paid employment had markedly lower relative risks of low growth mindset compared with retirees (RRR = 0.25, 95% CI: 0.19–0.33, *p* < .001), with similarly reduced risks among those in other non-retired employment categories (RRR = 0.57, 95% CI: 0.44–0.75, *p* < .001). Higher household wealth was also associated with a lower likelihood of a low-growth mindset. A higher memory score was associated with a lower likelihood of having a low growth mindset.

There was not enough evidence that ethnicity or urbanicity was associated with a low growth mindset.

##### High versus moderate growth mindset adult education

Sex, living arrangement, educational attainment, employment status, limiting longstanding illness, ethnicity, and urbanicity did not distinguish individuals with a high growth mindset from those with a moderate growth mindset. Older age and higher wealth, however, were indicative of differences: compared with those aged 50–59, participants in older age groups were progressively less likely to report a high growth mindset, and those in the highest wealth group were also less likely to report a high (rather than moderate) growth mindset.

##### Personality component of growth mindset

Table 2 presents the correlates of the proxy personality component of the growth mindset, explored simultaneously in the model.

**Table 2:** Correlates of the growth mindset-oriented personality component, England 2008–2012.

Variables	Low vs moderate		High vs moderate	
	RRR (95% CI)	*p*	RRR (95% CI)	*p*
**Sex**				
Men	Ref.		Ref.	
Women	1.28 (1.13–1.46)	<.001	1.03 (0.90–1.16)	.689
**Age group (years)**				
50–59	Ref.		Ref.	
60–69	0.97 (0.81–1.16)	.729	1.02 (0.87–1.20)	.807
70–79	1.04 (0.85–1.28)	.719	1.03 (0.84–1.26)	.777
80+	1.06 (0.80–1.40)	.681	0.98 (0.72–1.33)	.894
**Ethnicity**				
White	Ref.		Ref.	
Non-White	1.22 (0.79–1.88)	.379	0.98 (0.63–1.52)	.926
**Living alone**				
No	Ref.		Ref.	
Yes	1.04 (0.90–1.20)	.620	1.03 (0.89–1.21)	.673
**Education**				
≤14 years	Ref.		Ref.	
15–18 years	0.88 (0.71–1.09)	.232	0.93 (0.72–1.20)	.563
≥19 years	0.65 (0.50–0.85)	.002	1.27 (0.96–1.68)	.094
**Employment status**				
Retired	Ref.		Ref.	
In paid employment	0.97 (0.81–1.17)	.743	1.21 (1.00–1.45)	.050
Other^a^	1.02 (0.86–1.19)	.845	1.07 (0.91–1.27)	.411
**Wealth tertile**				
Lowest	Ref.		Ref.	
Middle	0.86 (0.74–0.99)	.040	0.91 (0.78–1.06)	.238
Highest	0.74 (0.63–0.87)	<.001	0.89 (0.76–1.05)	.164
**Area**				
Urban	Ref.		Ref.	
Rural	0.83 (0.72–0.96)	.011	0.95 (0.83–1.08)	.422
**Limiting illness**				
No (ref)	Ref.		Ref.	
Yes	1.20 (1.05–1.36)	.007	0.93 (0.81–1.06)	.272
**Memory score (continuous)**	0.95 (0.94–0.97)	<.001	1.05 (1.03–1.07)	<.001

*Note.* RRR = relative risk ratio. Estimates adjusted for nonresponse sampling weights.

a Permanently out of work, temporarily out of work, and looking after family.

##### Low versus moderate growth mindset personality

Women were significantly more likely than men to report a low personality-based growth mindset (RRR = 1.28, 95% CI: 1.13–1.46). Age was not significantly associated with the likelihood of reporting a low growth mindset compared to a moderate one. Ethnicity and living alone were not related to a higher relative risk of low growth mindset compared to medium. Educational attainment showed a clear protective effect: those who completed education at age 19 years or older were substantially less likely to report a low growth mindset compared with those who left school before age 14 (RRR = 0.65, 95% CI: 0.50–0.85). Employment status was not significantly associated with growth mindset. Higher household wealth and living in rural areas were associated with a lower likelihood of having a low-growth mindset. Participants with a limiting longstanding illness were more likely to report a low growth mindset (RRR = 1.20, 95% CI: 1.05–1.39), whereas higher memory scores were strongly associated with a lower likelihood of low growth mindset (RRR = 0.95 per unit increase, 95% CI: 0.94–0.97).

##### High versus moderate personality growth mindset

None of the characteristics revealed a significant likelihood of being in the high growth mindset group compared to moderate, with the exception of memory.

Higher memory scores were positively associated with a high growth mindset (RRR = 1.05 per unit increase, 95% CI: 1.03–1.07).

##### Beliefs component of growth mindset

Table 3 presents the correlates of the growth mindset-oriented beliefs component, explored simultaneously in the model.

**Table 3:** Correlates of the growth mindset-oriented belief component, England 2008–2012.

Variables	Low vs moderate		High vs moderate	
	RRR (95% CI)	*p*	RRR (95% CI)	*p*
**Sex**				
Men	Ref.		Ref.	
Women	0.89 (0.78–1.02)	.086	1.05 (0.93–1.18)	.442
**Age group (years)**				
50–59	Ref.		Ref.	
60–69	0.83 (0.68–1.00)	.050	1.08 (0.92–1.26)	.361
70–79	0.89 (0.72–1.10)	.286	0.86 (0.71–1.04)	.121
80+	1.28 (0.95–1.71)	.104	0.74 (0.55–1.00)	.046
**Ethnicity**				
White	Ref.		Ref.	
Non-White	0.84 (0.51–1.38)	.494	1.01 (0.66–1.54)	.961
**Living alone**				
No	Ref.		Ref.	
Yes	1.43 (1.23–1.66)	<.001	0.89 (0.76–1.03)	.122
**Education**				
≤14 years	Ref.		Ref.	
15–18 years	1.02 (0.81–1.29)	.854	1.03 (0.81–1.31)	.807
≥19 years	0.86 (0.65–1.15)	.311	1.16 (0.88–1.52)	.288
**Employment status**				
Retired	Ref.		Ref.	
In paid employment	0.71 (0.58–0.86)	.001	0.95 (0.80–1.14)	.596
Other^a^	0.98 (0.83–1.16)	.827	0.97 (0.82–1.14)	.716
**Wealth tertile**				
Lowest	Ref.		Ref.	
Middle	0.74 (0.64–0.87)	<.001	1.09 (0.93–1.26)	.278
Highest	0.61 (0.51–0.72)	<.001	1.47 (1.26–1.71)	<.001
**Area**				
Urban	Ref.		Ref.	
Rural	0.96 (0.83–1.12)	.623	1.07 (0.94–1.21)	.317
**Limiting illness**				
No (ref)	Ref.		Ref.	
Yes	2.22 (1.94–2.54)	<.001	0.61 (0.53–0.70)	<.001
**Memory score (continuous)**	0.98 (0.96–1.00)	.054	1.05 (1.03–1.07)	<.001

*Note*. RRR = relative risk ratio. Estimates adjusted for nonresponse sampling weights.

a Permanently out of work, temporarily out of work, and looking after family.

##### Low versus moderate growth mindset beliefs

There was not enough evidence that sex was associated with low growth mindset beliefs. Compared with those aged 50–59, participants aged 60–69 were less likely to report low beliefs (RRR = 0.83, 95% CI: 0.68–1.00, *p* = .050), while those aged 70–79 and 80+ did not differ significantly. Individuals living alone were substantially more likely to report low growth mindset beliefs compared with those not living alone (RRR = 1.43, 95% CI: 1.23–1.66). There was not enough evidence that educational attainment was associated with low beliefs. Being in paid employment was associated with a lower likelihood of low growth mindset beliefs compared with being retired (RRR = 0.71, 95% CI: 0.58–0.86), while other non-retired groups did not differ significantly. Household wealth showed a clear gradient: compared with the lowest tertile, those in the middle (RRR = 0.74, 95% CI: 0.64–0.87) and highest tertiles (RRR = 0.61, 95% CI: 0.51–0.72) were less likely to report low growth mindset beliefs. There was not enough evidence that ethnicity or urban-rural residence was associated with low beliefs. Participants with a limiting longstanding illness had a markedly higher likelihood of low growth mindset beliefs (RRR = 2.22, 95% CI: 1.94–2.54). Higher memory scores were associated with a lower likelihood of low growth mindset beliefs (RRR = 0.98 per unit increase, 95% CI: 0.96–1.00, *p* = .054), although this association was borderline.

##### High versus moderate growth mindset beliefs

Most individual characteristics were not associated with the likelihood of being in the high growth mindset beliefs group compared with the moderate group. The main exceptions were older age (80+), wealth, limiting longstanding illness, and memory. Compared with those aged 50–59, participants aged 80+ were less likely to report high growth mindset beliefs (RRR = 0.74, 95% CI: 0.55–1.00, *p* = .050), Household wealth was strongly associated: compared with the lowest tertile, those in the highest wealth tertile were more likely to report high growth mindset beliefs (RRR = 1.47, 95% CI: 1.26–1.71), while the middle tertile did not differ significantly. Having a limiting long-standing illness was associated with a lower likelihood of high growth mindset beliefs (RRR = 0.61, 95% CI: 0.53–0.70). Higher memory scores were positively associated with high growth mindset beliefs (RRR = 1.05 per unit increase, 95% CI: 1.03–1.07).

#### Affect grit

Table 4 presents the relative risk ratios (RRRs) and 95% confidence intervals (CIs) from a multinomial logistic regression model examining simultaneously sociodemographic and health-related correlates of grit-oriented affect. The reference category for the outcome was moderate grit.

**Table 4:** Correlates of the grit-oriented affect component, England 2008–2012.

Variables	Low vs moderate		High vs moderate	
	RRR (95% CI)	*p*	RRR (95% CI)	*p*
**Sex**				
Men	Ref.		Ref.	
Women	1.06 (0.93–1.21)	.357	1.19 (1.05–1.34)	.005
**Age group (years)**				
50–59	Ref.		Ref.	
60–69	0.67 (0.56–0.80)	.000	0.96 (0.81–1.12)	.592
70–79	0.61 (0.50–0.75)	.000	1.02 (0.84–1.25)	.813
80+	0.76 (0.57–1.02)	.065	0.86 (0.64–1.15)	.297
**Ethnicity**				
White	Ref.		Ref.	
Non-White	1.05 (0.66–1.68)	.836	1.24 (0.82–1.88)	.297
**Living alone**				
No	Ref.		Ref.	
Yes	1.25 (1.07–1.46)	.004	0.98 (0.85–1.14)	.797
**Education**				
≤14 years	Ref.		Ref.	
15–18 years	0.99 (0.78–1.25)	.939	0.90 (0.71–1.14)	.382
≥19 years	0.86 (0.65–1.14)	.302	1.05 (0.81–1.36)	.727
**Employment status**				
Retired	Ref.		Ref.	
In paid employment	0.79 (0.65–0.95)	.014	1.06 (0.89–1.27)	.518
Other^a^	0.85 (0.72–1.00)	.053	0.98 (0.83–1.15)	.800
**Wealth tertile**				
Lowest	Ref.		Ref.	
Middle	0.79 (0.68–0.92)	.003	1.06 (0.91–1.23)	.470
Highest	0.65 (0.55–0.78)	.000	1.17 (1.00–1.37)	.050
**Area**				
Urban	Ref.		Ref.	
Rural	0.85 (0.74–0.99)	.035	1.04 (0.92–1.19)	.522
**Limiting illness**				
No (ref)	Ref.		Ref.	
Yes	1.90 (1.66–2.17)	.000	0.78 (0.68–0.89)	.000
**Memory score (continuous)**	0.96 (0.94–0.98)	.000	1.02 (1.00–1.04)	.026

*Note*. RRR = relative risk ratio. Estimates adjusted for nonresponse sampling weights.

a Permanently out of work, temporarily out of work, and looking after family.

##### Low versus moderate affect grit

Participants aged 60–69 and 70–79 were significantly less likely to have low compared with moderate grit. Living alone was associated with a higher likelihood of low grit (RRR = 1.25, 95% CI: 1.07–1.46), as was reporting a limiting illness (RRR = 1.90, 95% CI: 1.66–2.17). Education, gender, and ethnicity were not significantly associated with low grit. Being in paid employment was linked to a lower relative risk of being in the low grit group (RRR = 0.79, 95% CI: 0.65–0.95). Higher wealth was associated with a lower relative risk of low grit: middle wealth tertile (RRR = 0.79, 95% CI: 0.68–0.92), highest wealth tertile (RRR = 0.65, 95% CI: 0.55–0.78). Living in a rural area was associated with slightly a lower relative risk of low grit (RRR = 0.85, 95% CI: 0.74–0.99). Better memory performance was associated with reduced relative risk of low grit (RRR = 0.96, 95% CI: 0.94–0.98).

##### High versus moderate affect grit

When comparing high versus moderate grit, few characteristics seemed to matter. For example, women were more likely than men to report high grit (RRR = 1.19, 95% CI: 1.05–1.34). Age, ethnicity, and living alone were not significantly associated with high grit. Higher wealth was modestly associated with a higher likelihood of high grit: highest tertile RRR = 1.17 (95% CI: 1.00–1.37), middle tertile not significant. Reporting a limiting illness was associated with a lower likelihood of high grit (RRR = 0.78, 95% CI: 0.68–0.89). Better memory performance was modestly but significantly associated with high grit (RRR = 1.02, 95% CI: 1.00–1.04).

#### Personality grit

Table 5 shows the correlates of the grit-oriented personality component, explored simultaneously in the model.

**Table 5:** Correlates of the grit-oriented personality component, England 2008–2012.

Variables	Low vs moderate		High vs moderate	
	RRR (95% CI)	*p*	RRR (95% CI)	*p*
**Sex**				
Men	Ref.		Ref.	
Women	0.80 (0.70–0.91)	.001	1.08 (0.95–1.22)	.236
**Age group (years)**				
50–59	Ref.		Ref.	
60–69	0.87 (0.72–1.06)	.159	0.88 (0.75–1.03)	.117
70–79	0.96 (0.78–1.19)	.735	0.85 (0.70–1.04)	.116
80+	0.97 (0.73–1.30)	.841	0.64 (0.47–0.86)	.004
**Ethnicity**				
White	Ref.		Ref.	
Non-White	1.03 (0.64–1.66)	.906	1.12 (0.74–1.69)	.599
**Living alone**				
No	Ref.		Ref.	
Yes	1.28 (1.10–1.50)	.002	0.96 (0.83–1.12)	.593
**Education**				
≤14 years	Ref.		Ref.	
15–18 years	0.92 (0.73–1.16)	.465	0.78 (0.61–0.99)	.042
≥19 years	1.00 (0.76–1.32)	.998	0.80 (0.61–1.05)	.103
**Employment status**				
Retired	Ref.		Ref.	
In paid employment	0.70 (0.58–0.85)	<.001	1.17 (0.98–1.40)	.091
Other^a^	0.91 (0.77–1.08)	.294	1.05 (0.89–1.24)	.587
**Wealth tertile**				
Lowest	Ref.		Ref.	
Middle	0.89 (0.76–1.04)	.135	1.14 (0.98–1.32)	.097
Highest	0.63 (0.53–0.75)	<.001	1.13 (0.96–1.32)	.136
**Area**				
Urban	Ref.		Ref.	
Rural	0.98 (0.85–1.13)	.784	0.98 (0.86–1.12)	.750
**Limiting illness**				
No (ref)	Ref.		Ref.	
Yes	1.98 (1.73–2.26)	<.001	0.57 (0.50–0.66)	<.001
Memory score (continuous)	0.95 (0.93–0.97)	<.001	1.00 (0.98–1.02)	.986

*Note.* RRR = relative risk ratio. Estimates adjusted for nonresponse sampling weights.

a Permanently out of work, temporarily out of work, and looking after family.

##### Low versus moderate personality grit

Women were significantly less likely than men to report low grit (RRR = 0.80, 95% CI: 0.70–0.91). Living alone was associated with a higher likelihood of low grit (RRR = 1.28, 95% CI: 1.10–1.50). Being in paid employment (RRR = 0.70, 95% CI: 0.58–0.85) and having higher wealth (RRR = 0.63, 95% CI: 0.53–0.75) were linked to a reduced risk of low grit relative to moderate grit. Participants reporting a limiting illness were substantially more likely to have low grit (RRR = 1.98, 95% CI: 1.73–2.26). Better memory performance was associated with a lower likelihood of low grit (RRR = 0.95, 95% CI: 0.93–0.97). Age, ethnicity, education, and living in rural areas were not significantly associated with low grit compared to medium grit.

##### High versus moderate personality grit

In the comparison of high versus moderate grit, most of the individual characteristics did not show significant associations. Age showed limited associations, though those aged 80+ were significantly less likely to report high grit (RRR = 0.64, 95% CI: 0.47–0.86). Participants with 15–18 years of education had a slightly lower likelihood of high grit compared with those with fewer than 14 years (RRR = 0.78, 95% CI: 0.61–0.99). Reporting a limiting illness was strongly associated with a lower likelihood of high grit (RRR = 0.57, 95% CI: 0.50–0.66).

Supplementary Tables 5–9 present comparisons of high versus low categories for each component. Across constructs, these analyses were broadly consistent with the main models: higher levels of growth mindset and grit were more likely among individuals with greater wealth, paid employment, and better memory performance, whereas older age (particularly 80+), living alone, and limiting longstanding illness were associated with a lower likelihood of belonging to the high group. These contrasts further emphasize the social and health patterning of lower psychological resources in later life.

Sensitivity analyses from linear regression models in which the Growth Mindset personality, Growth Mindset beliefs, Grit personality, and Grit affect components were analyzed using their continuous scores are presented in Supplementary Tables 10–13. The analyses yielded substantively similar patterns of association to those observed in the categorical analyses. In particular, socioeconomic position, limiting longstanding illness, and memory function remained consistently associated with the Growth Mindset and Grit components, supporting the robustness of the main conclusions.

## Discussion

Using nationally representative data on older adults in England, this study provides a population-level examination of Growth Mindset and Grit proxies in later life. By operationalizing proxy indicators across educational, personality, affective, and belief-based domains, we show that these constructs are not unitary traits but multidimensional psychological resources that are differentially distributed across socioeconomic position, health, and cognitive functioning. In doing so, we extend existing research beyond small or regionally specific samples and demonstrate that growth-oriented thinking and perseverance are socially patterned and embedded within cumulative life-course processes of advantage and disadvantage.

### Main findings

The distribution of the proxy components revealed important differences between domains. The adult education component of Growth Mindset was heavily skewed toward the low category, with almost four-fifths of participants reporting no engagement in formal learning over the previous four years. This measure may therefore capture access to, or engagement with, formal learning opportunities rather than growth-oriented behaviors more broadly. Older adults with a strong Growth Mindset may continue to seek personal development through other forms of learning and cognitive engagement, such as reading, hobbies, volunteering, cultural activities, or informal skill acquisition, which were not captured by the measure used in this study. In contrast, the personality and belief-based components of Growth Mindset showed more even distributions, with moderate and high scores well represented. Grit components showed a centrally skewed distribution peaking in the moderate category and broadly balanced distributions in the low and high categories. These results suggest that although formal educational activity becomes relatively uncommon in later adulthood, attitudinal, personality-based, and affective expressions of growth orientation and perseverance remain diverse in this population. This distinction underscores the importance of separating opportunity-dependent indicators from more dispositional or belief-based domains when studying psychological resources in older cohorts. It should be noted, however, that the categorization of personality and belief components into Low, Moderate, and High groups is based on ±0.5 *SD* thresholds, which are somewhat arbitrary and do not represent empirically derived cut-points. As such, these categories are intended to aid interpretation rather than indicate qualitatively distinct subgroups.

Age gradients were particularly marked for the education and beliefs components of Growth Mindset. Older participants were substantially more likely to fall into the low categories of these domains and less likely to be classified as high. This was confirmed in the regression models, which showed that each older age group had increasingly higher risks of low adult education Growth Mindset. The personality component showed only modest age differences, which is consistent with the idea that trait-based aspects of growth orientation may be more stable across the life course. This pattern aligns with life-course perspectives, suggesting relative stability in core personality traits alongside structural shifts in opportunity structures with advancing age.

Age effects on Grit were more nuanced. Older age groups were generally less likely to have low affect-based Grit, suggesting that emotional steadiness, determination, and positive affect related to perseverance may be relatively preserved. At the same time, the oldest participants were less likely to be in the high category of personality-based Grit, which may reflect accumulated health difficulties or reductions in the energy required for sustained goal pursuit. Such findings are consistent with theories of selective optimization with compensation, whereby goal persistence may be recalibrated in response to age-related constraints rather than simply diminished.

Gender differences were modest but consistent across some components. Women were less likely than men to fall into the low category of adult education Growth Mindset, but showed higher risks of low scores in the personality-based component. In contrast, women were less likely to have low personality-based Grit. These patterns may reflect gendered life-course experiences that shape how perseverance and growth-oriented beliefs are expressed, rather than inherent differences in psychological capacity. These findings are consistent with evidence that older women often display greater emotional resilience and social engagement, even when they have had fewer formal educational opportunities earlier in life (Phillips et al., 2016).

Living arrangements and health status emerged as central correlates for several components. Individuals living alone were more likely to fall into the low categories of the belief component of Growth Mindset and the low categories of both Grit components. Health status emerged as one of the most structurally consequential correlates across models. It was associated with substantially higher risks of low Growth Mindset and low Grit and markedly lower risks of high scores. These associations are consistent with studies showing that chronic illness and social isolation undermine perceived control, self-efficacy, and motivation in later life and reduce opportunities for cognitively and emotionally stimulating activity (Bandura, 1997; Dickens et al., 2011; Kempen et al., 2006).

Socioeconomic position, captured through educational attainment, employment status, and household wealth, was consistently associated with more favorable profiles. Completing full-time education at age 19 years or older was associated with lower risks of low Growth Mindset for some components, particularly the personality-based domain. Being in paid employment, compared with being retired, was associated with a lower likelihood of low adult education Growth Mindset, lower likelihood of low belief-based Growth Mindset, and reduced risks of low Grit in both affective and personality components. Household wealth showed particularly strong and consistent associations: higher wealth was linked to lower likelihoods of low Growth Mindset beliefs and low Grit, and to higher probabilities of belonging to the high categories of beliefs and Grit. Collectively, these findings suggest that Growth Mindset and Grit are socially patterned psychological resources, shaped by cumulative socioeconomic advantage across the life course. Rather than existing independently of structural conditions, these constructs appear embedded within material, educational, and occupational trajectories.

Cognitive performance also showed important associations with Growth Mindset and Grit. Higher memory scores were associated with more favorable profiles across most components, including lower likelihood of low Growth Mindset beliefs and low Grit, and higher likelihoods of high Growth Mindset beliefs and high Grit. Associations with the personality-based Growth Mindset component were more selective, but memory remained a consistent predictor of high scores across several domains. Although causal direction cannot be established, the observed associations raise the possibility of reciprocal relationships between cognitive functioning and growth-oriented psychological resources across the life course (Lachman & Agrigoroaei, 2010). The strong associations observed between personality-based components and memory performance are consistent with evidence that personality traits related to flexibility, persistence, and emotional regulation are robust correlates of cognitive functioning in older adulthood (Luchetti et al., 2016).

Ethnicity and area-level context played a more limited role. Ethnicity was not independently associated with most components. However, this finding should be interpreted cautiously given the relatively small proportion of ethnic minority participants in ELSA and the use of a broad White versus Non-White classification, which may have limited the ability to detect differences between ethnic groups.

### Comparisons with previous studies

Although direct comparisons with previous studies are limited by differences in measurement and study design, the present findings are in line with evidence suggesting that growth-oriented beliefs and behaviors tend to co-occur with more favorable cognitive, health, and social profiles in later life (Fabio et al., 2024; Lachman & Agrigoroaei, 2010). Our findings extend prior work by demonstrating that these associations are observable at the population level and persist even when constructs are operationalized using indirect indicators. However, the steep age gradients and high concentration of low scores observed, particularly for the adult education component, highlight the limitations of applying education-based Growth Mindset proxies to older populations. This likely reflects structural barriers to formal learning in later adulthood, rather than a lack of belief in personal growth. Studies of adult learning show that older adults often participate in informal learning rather than formal courses (Schuller & Watson, 2009), suggesting that measures of Growth Mindset in later life should be broadened to capture self- directed learning, technological adaptation, and everyday problem solving.

Our findings are also broadly consistent with studies showing associations between grit and more favorable cognitive, health, and social outcomes (Fleck et al., 2024; Kim & Lee, 2015; Rhodes & Giovannetti, 2022). The present analyses extend this literature by showing that different components of grit are shaped by different sociodemographic factors. For example, Rhodes and Giovannetti reported no association between grit and age, yet we found that the oldest participants were less likely to reach the highest levels of personality-based grit, although they were not overrepresented in the low category. This divergence may reflect differences in sample composition or cultural context and could indicate that the highest levels of sustained effort become harder to maintain in late life when health burdens accumulate.

A central contribution of this study is the demonstration that treating Growth Mindset and Grit as multidimensional constructs reveals heterogeneity that would remain obscured under single-trait conceptualizations. Much prior research has conceptualized Growth Mindset primarily as beliefs about the malleability of intelligence, as originally articulated in theories of implicit intelligence (Burnette et al., 2013; Dweck, 2006). These findings challenge intelligence-centered conceptualizations of Growth Mindset and suggest that growth orientation in later life may be better understood through belief systems, emotional regulation, and adaptive persistence in the face of structural and health-related constraints. Personality- and belief-based components displayed richer variation and stronger associations with social and health characteristics than the education-based component, which was heavily influenced by opportunity structures. Similarly, distinguishing between affective and personality-based grit highlighted important differences in the sociodemographic and health correlates of perseverance. These findings suggest that growth-oriented tendencies in later life may be expressed through multiple domains, including attitudes, beliefs, emotional resources, and behavioral engagement. While participation in formal learning was relatively uncommon in this cohort, future studies may benefit from incorporating a broader range of learning and cognitively stimulating activities to better capture how Growth Mindset is manifested in older adulthood.

### Strengths and limitations

A strength of this study is the use of a large, nationally representative cohort of older adults, which provides robust population-level estimates of how different aspects of Growth Mindset and Grit are distributed in later life. Furthermore, both constructs overlap conceptually with broader psychological resources, including perceived control, self-efficacy, openness to experience, and conscientiousness. The findings should therefore be interpreted as reflecting growth-oriented and perseverance-related tendencies rather than definitive measures of these constructs. Another possible limitation is that the measures used were proxy indicators rather than validated scales, and although they captured meaningful variation, they do not fully reflect the theoretical scope of Growth Mindset or Grit. In addition, some of the indicators overlap conceptually with related constructs, including perceived control, self-efficacy, openness to experience, conscientiousness, and positive affect. The findings should therefore be interpreted as reflecting growth-oriented and perseverance-related tendencies rather than definitive measures of these constructs. A further limitation relates to the categorization of continuous scores into low, moderate, and high groups. Although this approach facilitated interpretation and comparison across the different Growth Mindset and Grit proxy indicators, categorization may reduce statistical information and obscure variation within categories. However, supplementary analyses using the underlying continuous scores yielded substantively similar findings, suggesting that the main conclusions were robust to the modelling approach. Furthermore, although ELSA is a longitudinal study, the analyses were cross-sectional because the indicators used to derive the Growth Mindset and Grit proxy measures were not collected consistently across waves. In particular, the positive affect items used for the affective grit component were only available at Wave 5. Consequently, we were unable to examine longitudinal trajectories of these constructs, an important area for future research.

In conclusion, this study provides a population-level examination of proxy indicators related to Growth Mindset and Grit in later life. These indicators were socially patterned and associated with socioeconomic position, health, and cognitive functioning, suggesting that growth-oriented beliefs, learning-related behaviors, and perseverance-related tendencies are embedded within broader social and health contexts. By using data from a large, nationally representative cohort, this study extends previous research based largely on smaller and more selective samples and highlights the importance of considering psychological resources within a life-course and inequality framework. As populations age, a better understanding of how growth-oriented and perseverance-related tendencies relate to structural and social conditions may help inform strategies to support healthy aging.

## Supplementary Material

gbag134_Supplementary_Data

## Data Availability

The data that support the findings of this study are available from the UK Data Service (English Longitudinal Study of Ageing), subject to registration and standard access conditions. This study was preregistered on medRxiv: https://doi.org/10.64898/2026.02.27.26347198.
