# Supplementary material for: Growth mindset and grit as psychological resources in later life: age, socioeconomic status, and health patterning in the English Longitudinal Study of Ageing
Source: J Gerontol B Psychol Sci Soc Sci. 2026 Jul 11;81(10):gbag134. doi: 10.1093/geronb/gbag134 (PMC13589786; doi:10.1093/geronb/gbag134)
Supplement: Supplementary file 1 [file gbag134_supplementary_data.docx]

***The Journals of Gerontology, Series B: Psychological Sciences and Social Sciences* Supplementary Material: Zaninotto et al. Growth mindset and grit as psychological resources in later life: Age, socioeconomic, and health patterning in the English Longitudinal Study of Ageing.**

Below, we describe the operationalisation of each component of growth mindset and grit. The items included in each component are described below and in Supplementary Table 1. Because ELSA does not include validated Growth Mindset or Grit scales, we adopted a theory-informed proxy approach using conceptually related personality, belief, behavioural, and affective indicators available within the study. The selected indicators were not intended to reproduce the original scales developed by Dweck or Duckworth. Rather, they were chosen to capture distinct domains through which growth-oriented and perseverance-related tendencies may be expressed in later life. We therefore analyse these domains separately and interpret them as proxy indicators rather than direct measures of Growth Mindset or Grit.

***Growth Mindset Personality***

Personality was assessed at Wave 5 (2010-2011) using a short form of the Midlife Development Inventory (MIDI) (Lachman & Weaver, 1997), an instrument also employed in the US Health and Retirement Study. The questionnaire asked participants to rate how well a series of 26 descriptive adjectives described them, using a four-point Likert scale (1 = a lot, 4 =not at all). To capture trait-based tendencies conceptually related to growth-oriented attitudes, we constructed a personality index based on the following five items: Curious, Imaginative, Intelligent, Creative, and Broad-minded. To ensure that higher scores reflected greater endorsement of the trait, all items were reverse-coded. Each reverse-coded item was then standardised (z-scored) to place them on a common metric (mean = 0, SD = 1). A continuous growth-oriented personality score was constructed by averaging the five standardised items, such that higher values represented stronger endorsement of growth-related traits (Mean -0.000493; SD 0.69825; range -3.2876 to 1.3818) To aid interpretation, this continuous score was then categorised into three groups based on ±0.5 standard deviation thresholds: Low (< -0.5 SD), Moderate (-0.5 to 0.5 SD), and High (> 0.5 SD).

***Growth Mindset Beliefs***

A proxy measure of growth-oriented beliefs was operationalised using three items (“Chooses to do things they have never done”, “Feels that life is full of opportunities”, “Feels the future looks good to them”) from the CASP-19 (Hyde et al., 2003) quality of life questionnaire, administered at each ELSA wave, to capture attitudes consistent with growth-oriented perspectives. CASP19 has been administered at every wave via a self-completion questionnaire, with responses using a four-point Likert scale (1 = often, 4 =never). Responses were first recoded so that higher values reflected stronger endorsement of growth mindset beliefs. Each item was then standardised (z-scored) to place them on a common metric (mean = 0, SD = 1). A composite proxy index was created by averaging the three standardised items, yielding a continuous score of growth mindset belief (Mean -0.00128; SD 0.82654; range -2.4842 to 1.76625), with higher values representing stronger endorsement. The continuous score was categorised into three groups using ±0.5 standard deviation thresholds: Low (< -0.5 SD), Moderate (-0.5 to 0.5 SD), and High (> 0.5 SD).

***Growth Mindset Adult Learning***

Engagement in formal adult learning activities across waves was used as a behavioural proxy indicator of growth-oriented engagement with learning opportunities. At each wave, respondents were asked to report on their participation in formal learning experiences. Specifically, three items were used to capture continued learning and personal development: (1) whether they had obtained a further qualification in the past two years, (2) whether they had attended a formal course in the past month, and (3) whether they had participated in formal education or training in the past twelve months. These indicators reflect observable engagement in structured learning environments, which may signal an orientation toward continued learning and development. For this component, we extended the time window to include both Waves 4 and 5 of ELSA (2008-09 and 2010-12, respectively) rather than relying solely on Wave 5. This approach increases the likelihood of capturing infrequent or episodic learning behaviours that may not occur in a single wave, providing a more comprehensive measure of participants’ engagement in growth-oriented educational activity. It also reduces the risk of underestimating learning engagement due to the timing of data collection, ensuring that the resulting score better reflects participation in formal learning over time. A growth-oriented learning categorical variable was computed from the information of these variables as follows: Low (if they did not engage in any of the three learning activities in the 4 years prior the interview), Moderate (if they engaged in either a formal course or formal education training at least once in the 4 years prior the interview), High (if they obtained a further qualification in the 4 years prior the interview).

***Grit Personality***

Personality-based grit was operationalised using selected items from the Midlife Development Inventory (MIDI) personality questionnaire administered at Wave 5 (2010-11). This approach captures grit indirectly through stable personality characteristics, rather than using a dedicated grit scale.

The rationale for the selected traits is grounded in the core components of grit: perseverance, resilience, focus, and diligence. Specifically, the following MIDI items were chosen to reflect these dimensions: *Hardworking* and *Responsible* (perseverance of effort), *Calm* (resilience), *Organised* (focus), *Active* (commitment to goals), and *Thorough* (diligence/practice). These traits collectively capture an individual’s tendency to maintain effort and interest over long-term goals. Participants were asked to record how well each of the items described them, responses were recorded on a 5-point Likert scale ranging from 1 = “a lot” to 4 = “not at all.

Each item was reverse-coded where necessary so that higher values reflected stronger endorsement of grit-related characteristics. Items were then standardised (z-scored) to place them on a common metric (mean = 0, SD = 1), and a composite personality-based grit score was calculated by averaging the standardised items (Mean -0.000434; SD 0.675; range -2.952 to 1.196). Higher values indicate greater expression of grit. The continuous score was categorised into three groups based on ±0.5 standard deviation thresholds: Low (< -0.5 SD), Moderate (-0.5 to 0.5 SD), and High (> 0.5 SD).

***Grit Affect***

Affect-based grit was operationalised using selected positive affect items from a self-completion questionnaire administered in Wave 5 (2010-11). The questionnaire used measures of positive and negative affect chosen from the Positive and Negative Affect Schedule Expanded Form (Watson & Clark, 1994) also administered in the U.S. Health and Retirement Study. The items assess the frequency and intensity of both positive emotions (e.g., enthusiasm, alertness) and negative emotions (e.g., distress, irritability), providing a reliable overview of participants’ recent emotional experiences. Participants were asked to record to what degree in the past 30 days, they experienced 13 feelings, responses were recorded on a 5-point Likert scale ranging from 1 = “very much” to 5 = “not at all. Specifically, the following items were chosen to reflect these dimensions: *Determined*, *Hopeful*, *Attentive*, *Interested*, and *Inspired*. The rationale for the selected affects is grounded in the motivational and engagement aspects of grit. These positive affects collectively capture an individual’s tendency to maintain focus, energy, and optimism in the pursuit of long-term goals.

Each item was reverse-coded so that higher values reflected greater positive affect, indicative of stronger grit-related tendencies. Items were then standardised (z-scored) to place them on a common metric (mean = 0, SD = 1), and a composite affect-based grit score was calculated by averaging the standardised items (Mean -0.00210; SD 0.83; range -3.314 to 1.581). Higher values indicate greater expression of grit through positive affect. The continuous score was categorised into three groups based on ±0.5 standard deviation thresholds: Low (< -0.5 SD), Moderate (-0.5 to 0.5 SD), and High (> 0.5 SD).

**References**

Hyde, M., Wiggins, R. D., Higgs, P., & Blane, D. B. (2003, May). A measure of quality of life in early old age: the theory, development and properties of a needs satisfaction model (CASP-19). *Aging & Mental Health, 7*(3), 186-194. <https://doi.org/10.1080/1360786031000101157>

Lachman, M. E., & Weaver, S. L. (1997). *The Midlife Development Inventory (MIDI) Personality Scales: Scale construction and scoring*. Brandeis University. <https://www.brandeis.edu/psychology/lachman/pdfs/midi-personality-scales.pdf>

Watson, D., & Clark, L. A. (1994). Manual for the positive and negative affect schedule-expanded form. Iowa City, IA: The University of Iowa. <http://www.psychology.uiowa.edu/faculty/clark/panas-x.pdf>

**Supplementary Table 1. Components of each Growth Mindset and Grit Measure**

| **Construct** | **Source / Questionnaire** | **Wave(s)** | **Components / Items** | **Description** |
| --- | --- | --- | --- | --- |
| Growth Mindset Adult Learning | Main questionnaire  items | Waves 4 (2008-09) Wave 5 (2010-12) | Obtained a further qualification in the past two years  Attended a formal course in the past month  Participated in formal education or training in the past 12 months | Behavioural indicator of growth-oriented learning; captures participation in formal learning and personal development, reflecting an individual’s orientation toward continued learning and self-improvement |
| Growth Mindset Personality | Self-completion questionnaire using Midlife Development Inventory (MIDI) | Wave 5 (2010-11) | Curious  Imaginative  Intelligent  Creative  Broad-minded | Assesses trait-based tendencies consistent with openness to change and intellectual engagement; participants rated 26 adjectives on a 4-point Likert scale (1 = a lot, 4 = not at all) |
| Growth Mindset Beliefs | Self-completion questionnaire using CASP-19 quality of life questionnaire | Wave 5 (2012-13) | “Chooses to do things they have never done”  “Feels that life is full of opportunities”  “Feels the future looks good to them” | Operationalises growth mindset attitudes; CASP-19 administered via self-completion questionnaire at every wave, responses on a 4-point Likert scale (1 = often, 4 = never) |
| Grit Personality | Self-completion questionnaire using Midlife Development Inventory (MIDI) | Wave 5 (2010-11) | Hardworking and Responsible (perseverance of effort)  Calm (resilience)  Organised (focus)  Active (commitment to goals)  Thorough (diligence/practice) | Personality-based indicator of grit; selected items capture perseverance, resilience, focus, and diligence; participants rated how well each item described them on a 5-point Likert scale (1 = “a lot”, 4 = “not at all”) |
| Grit Positive Affect | Self-completion questionnaire using Positive and Negative Affect Schedule Expanded Form (PANAS-X) | Wave 5 (2010-11) | Determined  Hopeful  Attentive  Interested  Inspired | Affect-based indicator of grit; captures motivation, interest, and sustained engagement; participants reported frequency/intensity of emotions over past 30 days on a 5-point Likert scale (1 = “very much”, 5 = “not at all”) |

**Supplementary Table 2. Internal consistency of Growth Mindset and Grit proxy indicators**

| **Component** | **Number of items** | **Cronbach's α** |
| --- | --- | --- |
| Growth Mindset Personality | 5 | 0.732 |
| Growth Mindset Beliefs | 3 | 0.764 |
| Grit Personality | 6 | 0.750 |
| Grit Affect | 5 | 0.880 |

**Supplementary Table 3. Distribution of the components of the Growth Mindset and Grit scores, England 2008-2012**

|  | **Low**  **% (95%CI)** | **Moderate**  **% (95%CI)** | **High**  **% (95%CI)** |
| --- | --- | --- | --- |
| **Growth Mindset** |  |  |  |
| **Education** | 79.3 | 12.7 | 8.1 |
|  | (78.3; 80.2) | (11.9; 13.5) | (7.4; 8.8) |
| *N* | *5,971* | *976* | *566* |
| **Personality** | 26.3 | 49.9 | 23.8 |
|  | (25.2; 27.4) | (48.7; 51.1) | (22.8; 24.8) |
| *N* | *1,832* | *3,684* | *1,817* |
| **Beliefs** | 27.7 | 37.4 | 34.9 |
|  | (26.6; 28.9) | (36.2; 38.6) | (33.7; 36.1) |
| *N* | *1893* | *2763* | *2677* |
| **Grit** |  |  |  |
| **Personality** | 24.1 | 44.7 | 28.2 |
|  | (23.1; 25.2) | (46.5; 48.9) | (27.1; 29.3) |
| *N* | *1,669* | *3,558* | *2,106* |
| **Affect** | 26.7 | 44.5 | 28.8 |
|  | (25.6; 27.9) | (43.3; 45.7) | (27.7; 29.9) |
| *N* | *1,854* | *3,268* | *2211* |

Note: Estimates adjusted for non-response sampling weights

**Supplementary Table 4. Distribution of the components of the Growth Mindset and Grit scores according to age, England 2008-2012**

|  | **Low**  **Mean (s.e.)** | **Moderate**  **Mean (s.e.)** | **High**  **Mean (s.e.)** |
| --- | --- | --- | --- |
| **Growth Mindset Education** | 68.6 (0.15) | 62.4 (0.26) | 60.3 (0.26) |
| **Growth Mindset Personality** | 68.6 (0.27) | 67.0 (0.19) | 66.0 (0.26) |
| **Growth Mindset Beliefs** | 70.0 (0.29) | 66.9 (0.21) | 65.1 (0.18) |
| **Grit Personality** | 68.1 (0.29) | 67.1 (0.20) | 66.3 (0.22) |
| **Grit Affect** | 69.5 (0.29) | 67.0 (0.19) | 65.5 (0.24) |

Note: Estimates adjusted for non-response sampling weights. Sample size unweighted.

**Supplementary Table 5. Predictors of the growth mindset-oriented adult education component, England 2008-2012**

| **Predictor** | **High vs Low**  **RRR (95%CI)** | ***p*** |
| --- | --- | --- |
| **Sex** |  |  |
| Men | Ref. |  |
| Women | 1.03 (0.85; 1.26) | 0.760 |
| **Age group** |  |  |
| 50-59 | Ref. |  |
| 60-69 | 0.56 (0.45; 0.69) | <0.001 |
| 70- 79 | 0.17 (0.12; 0.26) | <0.001 |
| 80+ | 0.11 (0.05; 0.23) | <0.001 |
| **Ethnicity** |  |  |
| White | Ref. |  |
| Non-white | 1.03 (0.54; 1.95) | 0.933 |
| **Living alone** |  |  |
| No | Ref. |  |
| Yes | 1.04 (0.80; 1.36) | 0.754 |
| **Education** |  |  |
| ≤14 years | Ref. |  |
| 15-18 years | 0.84 (0.50; 1.40) | 0.499 |
| ≥19 years | 1.24 (0.72; 2.13) | 0.442 |
| **Employment status** |  |  |
| Retired | Ref. |  |
| In paid employment | 4.43 (2.96; 6.62) | <0.001 |
| Other† | 2.01 (1.34; 3.02) | 0.001 |
| **Wealth tertile** |  |  |
| Lowest | Ref. |  |
| Middle | 1.08 (0.85; 1.38) | 0.520 |
| Highest | 0.99 (0.76; 1.29) | 0.931 |
| **Area** |  |  |
| Urban | Ref. |  |
| Rural | 0.94 (0.76; 1.16) | 0.559 |
| **Limiting illness** |  |  |
| No | Ref. |  |
| Yes | 0.84 (0.66; 1.06) | 0.140 |
| **Memory score (continuous)** | 1.04 (1.01; 1.08) | 0.006 |

Note: Estimates adjusted for non-response sampling weights. †Permanently out of work, temporarily out of work, and looking after family.

**Supplementary Table 6. Predictors of the growth mindset-oriented personality component, England 2008-2012**

| **Predictor** | **High vs Low**  **RRR (95%CI)** | ***p*** |
| --- | --- | --- |
| **Sex** |  |  |
| Men | Ref. |  |
| Women | 0.80 (0.69; 0.93) | 0.004 |
| **Age group** |  |  |
| 50-59 | Ref. |  |
| 60-69 | 1.05 (0.86; 1.29) | 0.612 |
| 70-79 | 0.99 (0.78; 1.26) | 0.945 |
| 80+ | 0.92 (0.66; 1.30) | 0.646 |
| **Ethnicity** |  |  |
| White | Ref. |  |
| Non-white | 0.81 (0.48; 1.34) | 0.406 |
| **Living alone** |  |  |
| No | Ref. |  |
| Yes | 1.00 (0.84; 1.19) | 0.964 |
| **Education** |  |  |
| ≤14 years | Ref. |  |
| 15-18 years | 1.06 (0.81; 1.39) | 0.677 |
| ≥19 years | 1.96 (1.43; 2.68) | <0.001 |
| **Employment status** |  |  |
| Retired | Ref. |  |
| In paid employment | 1.24 (1.00; 1.55) | 0.050 |
| Other† | 1.06 (0.87; 1.28) | 0.575 |
| **Wealth tertile** |  |  |
| Lowest | Ref. |  |
| Middle | 1.06 (0.89; 1.27) | 0.496 |
| Highest | 1.20 (0.99; 1.45) | 0.058 |
| **Area** |  |  |
| Urban | Ref. |  |
| Rural | 1.14 (0.97; 1.34) | 0.124 |
| **Limiting illness** |  |  |
| No | Ref. |  |
| Yes | 0.77 (0.66; 0.91) | 0.001 |
| **Memory score (continuous)** | 1.10 (1.07; 1.12) | <0.001 |

Note: Estimates adjusted for non-response sampling weights. †Permanently out of work, temporarily out of work, and looking after family.

**Supplementary Table 7. Predictors of the growth mindset-oriented beliefs component, England 2008-2012**

| **Predictor** | **High vs Low**  **RRR (95%CI)** | ***p*** |
| --- | --- | --- |
| **Sex** |  |  |
| Men | Ref. |  |
| Women | 1.18 (1.02; 1.36) | 0.023 |
| **Age group** |  |  |
| 50-59 | Ref. |  |
| 60-69 | 1.30 (1.07; 1.58) | 0.007 |
| 70-79 | 0.97 (0.77; 1.21) | 0.768 |
| 80+ | 0.58 (0.42; 0.81) | 0.001 |
| **Ethnicity** |  |  |
| White | Ref. |  |
| Non-white | 1.20 (0.69; 2.10) | 0.521 |
| **Living alone** |  |  |
| No | Ref. |  |
| Yes | 0.62 (0.53; 0.73) | <0.001 |
| **Education** |  |  |
| ≤14 years | Ref. |  |
| 15-18 years | 1.01 (0.77; 1.32) | 0.952 |
| ≥19 years | 1.34 (0.98; 1.84) | 0.069 |
| **Employment status** |  |  |
| Retired | Ref. |  |
| In paid employment | 1.34 (1.09; 1.66) | 0.005 |
| Other† | 0.99 (0.82; 1.19) | 0.903 |
| **Wealth tertile** |  |  |
| Lowest | Ref. |  |
| Middle | 1.46 (1.24; 1.73) | <0.001 |
| Highest | 2.42 (2.02; 2.91) | <0.001 |
| **Area** |  |  |
| Urban | Ref. |  |
| Rural | 1.11 (0.95; 1.29) | 0.193 |
| **Limiting illness** |  |  |
| No | Ref. |  |
| Yes | 0.28 (0.24; 0.32) | <0.001 |
| **Memory score (continuous)** | 1.07 (1.05; 1.09) | <0.001 |

Note: Estimates adjusted for non-response sampling weights. †Permanently out of work, temporarily out of work, and looking after family.

**Supplementary Table 8. Predictors of the grit-oriented affect component, England 2008-2012**

| **Predictor** | **High vs Low**  **RRR (95%CI)** | ***p*** |
| --- | --- | --- |
| **Sex** |  |  |
| Men | Ref. |  |
| Women | 1.12 (0.97; 1.29) | 0.134 |
| **Age group** |  |  |
| 50-59 | Ref. |  |
| 60-69 | 1.43 (1.18; 1.73) | <0.001 |
| 70-79 | 1.68 (1.33; 2.12) | <0.001 |
| 80+ | 1.12 (0.81; 1.56) | 0.494 |
| **Ethnicity** |  |  |
| White | Ref. |  |
| Non-white | 1.18 (0.74; 1.89) | 0.478 |
| **Living alone** |  |  |
| No | Ref. |  |
| Yes | 0.78 (0.66; 0.93) | 0.006 |
| **Education** |  |  |
| ≤14 years | Ref. |  |
| 15-18 years | 0.91 (0.69; 1.19) | 0.490 |
| ≥19 years | 1.22 (0.89; 1.66) | 0.225 |
| **Employment status** |  |  |
| Retired | Ref. |  |
| In paid employment | 1.35 (1.09; 1.67) | 0.006 |
| Other† | 1.16 (0.96; 1.39) | 0.131 |
| **Wealth tertile** |  |  |
| Lowest | Ref. |  |
| Middle | 1.33 (1.13; 1.58) | 0.001 |
| Highest | 1.79 (1.49; 2.16) | <0.001 |
| **Area** |  |  |
| Urban | Ref. |  |
| Rural | 1.22 (1.04; 1.43) | 0.014 |
| **Limiting illness** |  |  |
| No | Ref. |  |
| Yes | 0.41 (0.35; 0.48) | <0.001 |
| **Memory score (continuous)** | 1.07 (1.04; 1.09) | <0.001 |

Note: Estimates adjusted for non-response sampling weights. †Permanently out of work, temporarily out of work, and looking after family.

**Supplementary Table 9. Predictors of the grit-oriented personality component, England 2008-2012**

| **Predictor** | **High vs Low**  **RRR (95%CI)** | ***p*** |
| --- | --- | --- |
| **Sex** |  |  |
| Men | Ref. |  |
| Women | 1.35 (1.16; 1.57) | <0.001 |
| **Age group** |  |  |
| 50-59 | Ref. |  |
| 60-69 | 1.01 (0.82; 1.24) | 0.912 |
| 70-79 | 0.89 (0.70; 1.13) | 0.326 |
| 80+ | 0.66 (0.47; 0.94) | 0.020 |
| **Ethnicity** |  |  |
| White | Ref. |  |
| Non-white | 1.09 (0.66; 1.80) | 0.748 |
| **Living alone** |  |  |
| No | Ref. |  |
| Yes | 0.75 (0.63; 0.89) | 0.001 |
| **Education** |  |  |
| ≤14 years | Ref. |  |
| 15-18 years | 0.85 (0.65; 1.12) | 0.245 |
| ≥19 years | 0.80 (0.58; 1.10) | 0.168 |
| **Employment status** |  |  |
| Retired | Ref. |  |
| In paid employment | 1.66 (1.33; 2.07) | <0.001 |
| Other† | 1.14 (0.94; 1.39) | 0.172 |
| **Wealth tertile** |  |  |
| Lowest | Ref. |  |
| Middle | 1.28 (1.07; 1.53) | 0.007 |
| Highest | 1.78 (1.46; 2.17) | <0.001 |
| **Area** |  |  |
| Urban | Ref. |  |
| Rural | 1.00 (0.85; 1.18) | 0.994 |
| **Limiting illness** |  |  |
| No | Ref. |  |
| Yes | 0.29 (0.25; 0.34) | <0.001 |
| **Memory score (continuous)** | 1.05 (1.03; 1.08) | <0.001 |

Note: Estimates adjusted for non-response sampling weights. †Permanently out of work, temporarily out of work, and looking after family.

**Supplementary Table 10. Linear regression of continuous Growth Mindset Beliefs score, England 2008-2012**

| **Predictor** | **β coefficient (95% CI)** | ***p*** |
| --- | --- | --- |
| **Sex** |  |  |
| Men | Ref. |  |
| Women | 0.036 (-0.004; 0.076) | 0.081 |
| **Age group** |  |  |
| 50-59 | Ref. |  |
| 60-69 | 0.077 (0.026; 0.128) | 0.003 |
| 70-79 | 0.009 (-0.054; 0.072) | 0.786 |
| 80+ | -0.191 (-0.287; -0.096) | <0.001 |
| **Ethnicity** |  |  |
| White | Ref. |  |
| Non-White | 0.019 (-0.146; 0.184) | 0.822 |
| **Living alone** |  |  |
| No | Ref. |  |
| Yes | -0.150 (-0.201; -0.099) | <0.001 |
| **Education** |  |  |
| ≤14 years | Ref. |  |
| 15-18 years | 0.010 (-0.070; 0.091) | 0.805 |
| ≥19 years | 0.094 (0.004; 0.183) | 0.040 |
| **Employment status** |  |  |
| Retired | Ref. |  |
| In paid employment | 0.091 (0.033; 0.149) | 0.002 |
| Other† | 0.012 (-0.043; 0.067) | 0.678 |
| **Wealth tertile** |  |  |
| Lowest | Ref. |  |
| Middle | 0.129 (0.079; 0.178) | <0.001 |
| Highest | 0.244 (0.194; 0.294) | <0.001 |
| **Area** |  |  |
| Urban | Ref. |  |
| Rural | 0.025 (-0.018; 0.067) | 0.249 |
| **Limiting illness** |  |  |
| No | Ref. |  |
| Yes | -0.434 (-0.478; -0.390) | <0.001 |
| **Memory score (continuous)** | 0.021 (0.014; 0.027) | <0.001 |

Note: Estimates adjusted for non-response sampling weights. †Permanently out of work, temporarily out of work, and looking after family.

**Supplementary Table 11. Linear regression of continuous Growth Mindset Personality score, England 2008-2012**

| **Predictor** | **β coefficient (95% CI)** | ***p*** |
| --- | --- | --- |
| **Sex** |  |  |
| Men | Ref. |  |
| Women | -0.069 (-0.105; -0.034) | <0.001 |
| **Age group** |  |  |
| 50-59 | Ref. |  |
| 60-69 | 0.005 (-0.042; 0.052) | 0.826 |
| 70-79 | -0.014 (-0.071; 0.043) | 0.635 |
| 80+ | -0.019 (-0.103; 0.065) | 0.658 |
| **Ethnicity** |  |  |
| White | Ref. |  |
| Non-white | -0.084 (-0.222; 0.053) | 0.231 |
| **Living alone** |  |  |
| No | Ref. |  |
| Yes | -0.010 (-0.054; 0.035) | 0.675 |
| **Education** |  |  |
| ≤14 years | Ref. |  |
| 15-18 years | 0.043 (-0.028; 0.115) | 0.236 |
| ≥19 years | 0.178 (0.097; 0.259) | <0.001 |
| **Employment status** |  |  |
| Retired | Ref. |  |
| In paid employment | 0.061 (0.009; 0.114) | 0.021 |
| Other† | 0.010 (-0.038; 0.058) | 0.673 |
| **Wealth tertile** |  |  |
| Lowest | Ref. |  |
| Middle | 0.027 (-0.017; 0.071) | 0.222 |
| Highest | 0.069 (0.023; 0.114) | 0.003 |
| **Area** |  |  |
| Urban | Ref. |  |
| Rural | 0.029 (-0.008; 0.066) | 0.130 |
| **Limiting illness** |  |  |
| No | Ref. |  |
| Yes | -0.071 (-0.110; -0.032) | <0.001 |
| **Memory score (continuous)** | 0.025 (0.019; 0.030) | <0.001 |

Note: Estimates adjusted for non-response sampling weights. †Permanently out of work, temporarily out of work, and looking after family.

**Supplementary Table 12. Linear regression of continuous Grit Affect score, England 2008-2012**

| **Predictor** | **β coefficient (95% CI)** | ***p*** |
| --- | --- | --- |
| **Sex** |  |  |
| Men | Ref. |  |
| Women | 0.045 (0.003; 0.088) | 0.034 |
| **Age group** |  |  |
| 50–59 | Ref. |  |
| 60–69 | 0.107 (0.050; 0.163) | <0.001 |
| 70–79 | 0.169 (0.101; 0.237) | <0.001 |
| 80+ | -0.005 (-0.110; 0.099) | 0.924 |
| **Ethnicity** |  |  |
| White | Ref. |  |
| Non-white | 0.047 (-0.109; 0.204) | 0.553 |
| **Living alone** |  |  |
| No | Ref. |  |
| Yes | -0.102 (-0.157; -0.047) | <0.001 |
| **Education** |  |  |
| ≤14 years | Ref. |  |
| 15–18 years | -0.046 (-0.133; 0.040) | 0.294 |
| ≥19 years | 0.018 (-0.079; 0.114) | 0.721 |
| **Employment status** |  |  |
| Retired | Ref. |  |
| In paid employment | 0.101 (0.039; 0.162) | 0.001 |
| Other† | 0.030 (-0.027; 0.087) | 0.301 |
| **Wealth tertile** |  |  |
| Lowest | Ref. |  |
| Middle | 0.125 (0.073; 0.176) | <0.001 |
| Highest | 0.201 (0.147; 0.254) | <0.001 |
| **Area** |  |  |
| Urban | Ref. |  |
| Rural | 0.053 (0.008; 0.097) | 0.020 |
| **Limiting illness** |  |  |
| No | Ref. |  |
| Yes | -0.317 (-0.363; -0.271) | <0.001 |
| **Memory score (continuous)** | 0.019 (0.012; 0.025) | <0.001 |

Note: Estimates adjusted for non-response sampling weights. †Permanently out of work, temporarily out of work, and looking after family.

**Supplementary Table 13. Linear regression of continuous Grit Personality score, England 2008-2012**

| **Predictor** | **β coefficient (95% CI)** | ***p*** |
| --- | --- | --- |
| **Sex** |  |  |
| Men | Ref. |  |
| Women | 0.073 (0.040; 0.107) | <0.001 |
| **Age group** |  |  |
| 50–59 | Ref. |  |
| 60–69 | 0.009 (-0.033; 0.051) | 0.685 |
| 70–79 | -0.005 (-0.057; 0.048) | 0.858 |
| 80+ | -0.089 (-0.173; -0.005) | 0.037 |
| **Ethnicity** |  |  |
| White | Ref. |  |
| Non-white | 0.014 (-0.123; 0.151) | 0.843 |
| **Living alone** |  |  |
| No | Ref. |  |
| Yes | -0.081 (-0.125; -0.038) | <0.001 |
| **Education** |  |  |
| ≤14 years | Ref. |  |
| 15–18 years | -0.002 (-0.075; 0.072) | 0.963 |
| ≥19 years | -0.020 (-0.101; 0.060) | 0.621 |
| **Employment status** |  |  |
| Retired | Ref. |  |
| In paid employment | 0.158 (0.108; 0.209) | <0.001 |
| Other† | 0.051 (0.003; 0.099) | 0.039 |
| **Wealth tertile** |  |  |
| Lowest | Ref. |  |
| Middle | 0.086 (0.044; 0.128) | <0.001 |
| Highest | 0.139 (0.096; 0.182) | <0.001 |
| **Area** |  |  |
| Urban | Ref. |  |
| Rural | 0.002 (-0.033; 0.038) | 0.897 |
| **Limiting illness** |  |  |
| No | Ref. |  |
| Yes | -0.313 (-0.351; -0.275) | <0.001 |
| **Memory score (continuous)** | 0.015 (0.010; 0.021) | <0.001 |

Note: Estimates adjusted for non-response sampling weights. †Permanently out of work, temporarily out of work, and looking after family.
